# Interactive knowledge discovery with the doctor-in-the-loop: a practical example of cerebral aneurysms research

**DOI:** 10.1007/s40708-016-0038-2

**Published:** 2016-02-24

**Authors:** Dominic Girardi, Josef Küng, Raimund Kleiser, Michael Sonnberger, Doris Csillag, Johannes Trenkler, Andreas Holzinger

**Affiliations:** 1 RISC Software GmbH - Research Unit Medical Informatics, Johannes Kepler University Linz, Hagenberg, Austria; 2 Institute for Application Oriented Knowledge Processing, Johannes Kepler University Linz, Linz, Austria; 3 Institute for Radiology, Campus Neuromed of the Medical University Linz, Linz, Austria; 4 Research Unit HCI-KDD, Institute for Medical Informatics, Statistics and Documentation, Medical University Graz, Graz, Austria

**Keywords:** Doctor-in-the-loop, Expert-in-the-loop, Interactive machine learning, Process model, Knowledge discovery, Medical research

## Abstract

Established process models for knowledge discovery find the domain-expert in a customer-like and supervising role. In the field of biomedical research, it is necessary to move the domain-experts into the center of this process with far-reaching consequences for both their research output and the process itself. In this paper, we revise the established process models for knowledge discovery and propose a new process model for domain-expert-driven interactive knowledge discovery. Furthermore, we present a research infrastructure which is adapted to this new process model and demonstrate how the domain-expert can be deeply integrated even into the highly complex data-mining process and data-exploration tasks. We evaluated this approach in the medical domain for the case of cerebral aneurysms research.

## Introduction

Clinical researchers today are confronted with increasingly large, complex, and high-dimensional datasets [1]. Consequently, the application of interactive visual data exploration in combination with machine-learning techniques for knowledge discovery and data mining is indispensable. However, these algorithms work well in lower-dimensional spaces and well-defined environments, but in the biomedical domain, we are confronted with probability, uncertainty, incompleteness, vagueness, noise, etc., which make the application of automated approaches difficult, and the complexity of machine-learning algorithms have kept away non-computing experts from the application of such solutions in their daily research workflow. These clinical researchers, or domain-experts are usually no computing experts. They have highlevel medical domain-expert knowledge to perform their research, to interpret newly gained knowledge and patterns in their data, but in practice rather only have basic or rudimentary computation know-how. A smooth interaction of the domain-expert with the data would greatly enhance the whole knowledge-discovery process chain [2]. In daily clinical research, the actual process differs significantly from the established process descriptions. In the commonly known definitions (see [3] for a good overview), the domain-expert is seen in a supervising, consulting, and a customer role: a person who is outside the process and assists in crucial aspects with domain knowledge and receives the results. All the other steps of the process are performed by the so-called data analysts, who are supported by the domain-experts in understanding for the current research project the relevant aspects of the research domain and in interpreting the results. However, for the analysis of medical data, detailed and explicit medical expert knowledge and knowledge of clinical processes is urgently required. Roddick et al. [4] point out that data mining in medical domain requires significant domain-expertise and cannot be performed without the intense cooperation of medical domain-experts. This clearly distinguishes the data mining in the medical domain from data mining in the market basket or financial-trading data. Furthermore, Roddick et al. suggest that the findings of data mining in medical research should only be interpreted as suggestions for further research. Cois and Moore [5] stress the uniqueness of medical data mining, caused by the nature of its data and other aspects. This is also supported by Bellazi and Zupan [6], who stress the safety aspect of medical knowledge discovery, which is an often neglected part, as the expert-in-the-loop (in the biomedical sciences, we speak of a “doctor-in-the-loop”) is a new paradigm in information-driven medicine, seating the expert as authority inside a loop supplying him/her with information on the actual patient data [7].

The integration of the domain-expert directly into data exploration and data mining tasks is a relatively recent approach, and it should be emphasized that data mining is only one step of the whole interactive knowledge-discovery process chain (see Fig. 2 in [2]). Consequently, it is mandatory to investigate which tasks arise for the domain-experts as central actors of the whole knowledge-discovery process, and what consequences this paradigm shift has for the process itself. In this paper, we focus on aspects of a novel, process model. We also present an ontology-based research-data infrastructure for medical research which is based upon the newly presented process model for knowledge discovery. Furthermore, we will show by a concrete example how this generic infrastructure is used in everyday clinical research. We also show how the elaborated structural meta-information of the domain ontology is used to lower the technical barriers for medical domain-experts to use advanced visualization and data-mining algorithms.

## Related research

There is not considerable amount of research as yet on this hot topic. A reason for sure is that the term “interactive knowledge discovery” is not a well-established or clearly defined term.

A recent work from 2014 by Mirchevska et al. [8] presents a method for combining domain knowledge and machine learning for classifier generation and online adaptation, which exploits advantages in domain knowledge and machine learning as complementary information sources. The authors state that while machine-learning methods may discover patterns in domains that are too subtle for humans to detect, domain knowledge of an expert may contain information on a domain not even present in the available domain data. This aspect may have huge influence on medical research.

A good example for interactive knowledge discovery is the work by Mueller et al. [9] where. in the data-mapping phase, which is done by a biomedical expert, the data attributes of the meta-information are compared with the visual capabilities of the graphical elements in order to give a feedback to the user about the correctness of the variable mapping.

In 2007, Inokuchi et al. [10] described MedTAKMI-CDI, an online analytical processing system, which enables the interactive discovery of knowledge for clinical decision intelligence (CDI) which supports decision making by providing in-depth analysis of clinical data from multiple sources on a database of about 7000 patients at the National Cancer Center in Japan.

The essence is that the elicitations of knowledge from domain-experts and empirical machine learning are two distinct approaches for knowledge discovery with different and mutually complementary capabilities [11].

### Established process models

In 1996, Usama Fayyad, Gregory Piatetsky-Shapiro, and Padhraic Smyth published a number of articles [12], [13, 14] which build the base for what we call now the process of knowledge discovery in databases. Soon, further process models were published with different focuses, degrees of detail [15, 16], and so on. In general, there is a huge consensus among these process models. In their review paper in 2006, Kurgan et al. [3, Table 1 on page 6] even managed to extract a generic process model out of the previous, most-established process models.

Aside from the significant consensus concerning the steps of these process models, there is also a huge agreement about the roles within these processes. The process is executed by a so-called data analyst, a person whose profile varies from that of a computer scientist, to that of statistician or data-mining expert. The domain-expert is always seen in an external position, as a customer and/or supervisor. This fact is clearly reflected by the first steps of the generic process model (and hence of most other process models): *1—Understanding the Domain* and *2—Understanding the Data*. Both steps would be unnecessary for domain-experts within the process loop.

## A new process model

### Proposal

Keeping in mind that medical domain-experts are required to be deeply involved into the process of medical knowledge discovery [5], the known process models are hardly suitable. A new process model is needed, which regards the central role of the domain-experts.

**Fig. 1:**
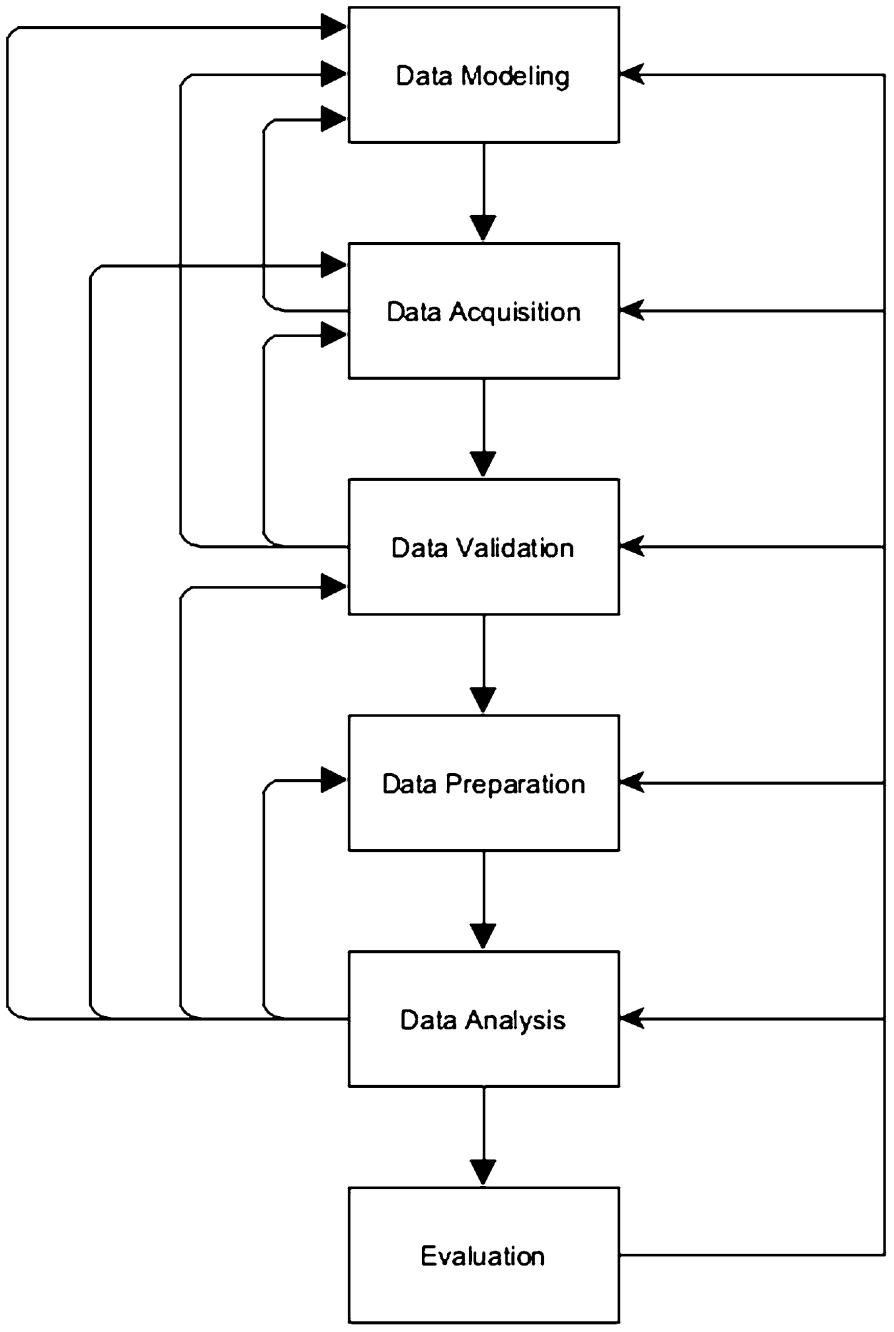
A new process model for domain-expert-centered knowledge discovery in biomedical research

We present a new process model for domain-expert-centered knowledge discovery in biomedical research—see Fig. 1. It is, of course, closely related to and derived from the existing models, but differs in crucial aspects. The major difference to the established definitions cannot be seen in this process description, as it takes place at another level. It is the role of the medical domain-experts switched from the edge of the process to the center. Subsequently, the first significant difference is the absence of the step, ‘Understanding of the Problem‘, which is of course caused by the new major player of the process, who does no longer need to invest time in getting into the research matter. Hence, the steps of the new process are defined as follows:

1. *Data modeling* This step is closely related to the step, ‘Understanding of the Data,‘ in the definitions of [17]. It is necessary for the researcher(s) to be aware of what kinds of data are needed to be able to answer the research questions: Which data entities from my research domain are relevant for the current research projects? Which of their attributes are needed? and In what kind of relations are they in? This term is closely related to the term *Experimental Design* with the difference that experimental design is a kind of super set of data modeling. Data modeling can be a part of the experimental design, but it only focuses on the structure and validity rules of the research data that need to be stored. Experimental design usually regards further aspects, such as the desired sample sizes, inclusion and exclusion criteria, the schedule, etc. as well. This data definition, which will be called the domain ontology from now on, builds the base for all further data-based operations, and differs from one research project to another and from one domain to another. This distinguishes this process definition from many conventional definitions, where only available data—data which are produced in everyday routines—are analyzed. In order to be able to answer medical research questions, it is necessary to overcome the bias of using only what is easily available.
2. *Data acquisition* Especially in medical, scientific research, it is often necessary to acquire the needed . Data which are stored in electronic hospital information systems (HIS) are hardy suitable for scientific research because they often contain semi-structured, textual data [18], or the data mostly used for billing and documentation purposes [19]. Especially medical diagnoses and interpretations of medical test are often stored as free text. Furthermore, redundant and contradictory data also occur. Although data mining has already been performed directly on HIS, its results are less scientifically applicable than for management purposes [20, 21]. The missing or the lack of reusable data stored in clinical information system has already been identified as a major challenge to medical informatics [22].
3. *Data validation* The quality of the outcome of a research projects strongly depends on the quality of the underlying data. As already mentioned above, data quality is a widely underestimated issue in medical datasets, and even data from electronic sources (hospital information systems, etc.) are erroneous and inconsistent. Considering the complexity and the huge amount of medical data needed for medical research, the need for an automatic data validation becomes obvious. Data quality is known to be a generally underrepresented topic in medical publications [23].
4. *Data preparation* Data analysis is rarely performed directly on the whole dataset. Usually, datasets of interest are created for certain hypotheses, and erroneous or implausible data are removed from these sets. Furthermore, in medicine, very often changes or differences (functions on data values), rather than raw data, contain valuable information [4]. Consequently, it is necessary to define these desired functions on the data and make their results accessible to researchers/users as new calculated variables.
5. *Data analysis* In this phase, the actual step of knowledge discovery is performed, using either conventional statistics or methods of data mining, machine learning, or means of visual analytics.
6. *Evaluation* In this final step, the gained knowledge must be clinically evaluated and verified.

The steps of this process are not necessarily aligned to follow a strict sequence. On the one hand, steps happen in parallel or strongly overlap with each other. Hence, it is possible to see the steps of data acquisition and data validation follow a sequential order, where validation is performed as soon as all the data are acquired. Alternatively, data acquisition and data validation can occur in parallel where each newly entered item of data is immediately verified. Furthermore, it is of course possible to perform data preparation and subsequent analysis alongside, while the data acquisition is still in progress. On the other hand, there exist a number of feedback loops, such as from almost any step of the process to data modeling. This means at any of these steps, it may become necessary to adapt the actual domain ontology. Furthermore, insights gained from data validation and data analysis may require reacquisition or revision of the existing data. And results from first data analysis may reveal systematic data errors, which entail a revision of the data-validation rules or the data-preparation algorithms.

### Consequences and challenges

The researching medical domain-experts face a number of challenges and obstacles when they try to perform medical research and knowledge discovery. The situation is worsened by the fact that research projects with limited funding often complete lack an explicit IT support. So the researchers find themselves in a situation where they have to deal with both, the complexity of their research domain and the complexity of their own data and data structures with all its consequences.

The selection, setup, and maintenance of a research data infrastructure have already been identified as a major obstacle in biomedical research [24]. In 2007, a survey among biomedical researchers [25] found out that data handling in general had become a major barrier in a number of biomedical research projects. Furthermore, biomedical researchers are often hardly able to cope with the complexity of their own data. The fact that many researchers use general-purpose office applications, which do not provide any support in data handling, worsens the situation.

Although highly sophisticated data mining (DM) and machine-learning (ML) algorithms have been used in other domains for decades, their usage in the field of medical research is still limited. A survey from 2012 among hospitals from South Africa, Germany, Switzerland, Lithuania, and Albania [26] showed that only 29 % of the medical personnel of responders were familiar with a practical application of DM. Although the survey is sure not globally representative, it indicates that medical research is still widely based on basic statistical methods, which might be sufficient in low-dimensional settings, but medical data tend to be high dimensional. One reason for this rather low acceptance rate is the relatively high technical obstacle that needs to be taken in order to apply these algorithms combined with the limited knowledge about the algorithms themselves and their output. A view that is shared by [27] who states that ’*the grand challenge is to combine these diverse fields to support the expert end users in learning to interactively analyze information properties thus enabling them to visualize the relevant parts of their data*’.

Since the medical domain itself is a very complex one and data acquisition is usually done by multiple persons over a certain period of time, it is crucial for subsequent data analysis to check the plausibility and validity of the collected data. Simple recording errors can usually be detected by simple rules, but systematic and procedural errors, which are known to cause severe bias to the study outcome [28], can rather be detected by high complex rules. In general, data quality in medical research project is not a well-researched topic [23].

## Application and implementation

In order to address all these challenges we developed a generic, ontology-centered research infrastructure. The main principle is the following: By modeling the actual research domain in the form of a domain ontology (Step 1 of the process), the domain-experts build the base for all subsequent steps. The whole research infrastructure derives its structure and behavior from the central domain ontology—at run-time. Changes to the ontology have immediate effects on the whole system, which consists of three main modules. Firstly, a management tool, which allows the user to model and maintain the domain ontology, but also process and analyze the research data. The other two components are an ontology-derived electronic data interface based upon and open-source ETL (Extract-Transform-Load) suite, and an ontology-derived web interface for manual data input and processing. Wherever possible the elaborate structural meta-information is used to actively support the user in data handling, processing and analyzing. The system always appears to the user as if it was especially tailored for his domain. For further information in more detail on the infrastructure itself, the reader is kindly referred to [29, 30, 31].

Based on one particular example, we now want to show how this process in combination with an appropriate software system can enable the domain-expert to utilize advanced visualization methods and machine-learning algorithms.

### Ontology-guided meta-classification

Given the following situation: The researcher used the above-mentioned research infrastructure for collecting his research data and now wants to investigate the influences (possibly nonlinear) of a number of features on a target class. Experts in the field of computer science will recognize this problem as a binary classification problem. In order to answer this question to the researcher, the following approach was made: After the user selects the potential features and the desired target class for a given dataset, a number of classification algorithms in numerous configurations are launched in parallel in the background. The whole data transformation and pre-processing are performed automatically by means of the extensive structural meta-information available from the current domain ontology. For all resulting classification models a tenfold cross validation is performed and the area under the RoC curve of each classification algorithm and configuration is calculated. As a result, the best area under RoC of each algorithm are consolidated and presented in a user-friendly way. In this way the research gets an indication whether the assumed influence is measurable or not. This approach is based upon the following assumptions:

1. The quality of the classification model that is developed by a classification algorithm in a reasonable (default) configuration or in an automatically optimized configuration provides an indication as to whether a reliable classification is possible at all, or not; for example. if such a classification model shows an area under the ROC curve of something close to 0.5, then it is rather unlikely to increase the quality of the classification model to a satisfying level just by adjusting and tuning the algorithms’ parameters. The more promising way is to adjust the input set of input variables.
2. If none of the applied classification algorithms in any of the used configuration is able to yield a satisfying classification model then it is assumed that there is no measurable influence of the input features on the target class within the available dataset.

It has to be kept in mind, that this approach shows a number of limitations and restrictions: The yielded result is an indication whether an influence can be assumed, not a classification model. The models themselves are only a means to get a result. The result does not provide any information an statistical significance of the discovered phenomena. The result does not provide any information on causalities and reasons for the discovered phenomena. This approach has yet to take into account the correlations among the input features. This approach has yet to provide any information whether a subset of the chosen features would have been sufficient to predict the class label. This approach does not provide any explanation component on how strong or in which way the features influence the target class. Nonetheless, it does yield an easy-to-use and easy-to-interpret indication on whether the assumed (even nonlinear) influence can be measured in the data.

For a first test setup, the following algorithms were used: A Naive Bayes classifier, a Random Forest, a Logistic Regression, a Support Vector Machine with Grid Search optimization [32], and a Multi-Layer Perceptron. The ontology-guided meta-classifier was tested using a number of generated and publically available datasets with promising results (see [33]) and will now be tested on actual clinical research data.

### Ontology-guided dimensionality reduction for visual analytics

It is an often re-occurring requirement in medical research to find groups of similar elements, e.g., patients with similar symptoms or anamnesis. This process is often referred to as clustering or unsupervised learning. Cluster analysis is defined as the organization of a collection of patterns (usually represented as a vector of measurements, or a point in a multidimensional space) into clusters based on similarity [34]. Cluster algorithms try to find groups of similar records and group them into meaningful clusters. The cluster membership of each data record is usually marked with a cluster number or cluster label. Without any visual check the result of the clustering is very hard to interpret. It provides no information one shape of each cluster and no information of the topology among the clusters. Although cluster analysis is an established state-of-the-art method, its direct benefit for the domain-expert is very limited.

In order to overcome these drawbacks of classical cluster algorithms the decision was made to follow a visual analytics paradigm. Therefore, the potentially high-dimensional research data need to be mapped onto a two-dimensional display. Two well-known algorithms for these tasks are the Self-Organizing or Kohonen Map (SOM) [35] and the nonlinear mapping algorithm of Sammon’s mapping [36]. Both algorithms try to minimize the error or mismatch between topologies in the n-dimensional source-space and the (mostly) two-dimensional target space.

For the medical researcher, the nonlinear mapping algorithm is hidden behind the notion ’Visual Clustering.’ The only configuration, which is required by the user, is the selection of attributes that should be taken into account for the calculation of the distance or dissimilarity of two records. Then, the algorithm normalizes the data. Subsequently, a distance matrix is calculated, whereas for the numerical variables, an Euclidean distance (after normalization) is used and an extension of the well-known Jaccard Metric for categorical variables. The Jaccard Metric was extended in a way that it takes into consideration the similarity of categorical concepts if they are organized in a hierarchical structure, e.g., the ICD-10 catalog. The similarity is determined base on the relative position of concepts in the concept hierarchy. A publication on this extension is in progress. Finally, the result is presented in a scatter plot. By means of a mouse wheel, the user is able to change the variable that is used to color the dots. In this way, not only patterns in the topology of the data can be identified but also the correlation to other attributes according to the coloring. Within the plot, the user is able to select datasets of interest and directly access and process the underlying data records. Plots showing the same set of interest are linked with each other in such a way that the selection on one plot is automatically highlighted on all other plots as well.

### Clinical application

#### The aneurysm registry

A medical registry is a systematic collection of a clearly defined set of health and demographic data for patients with specific health characteristics, held in a central database for a predefined purpose [37]. The aneurysm registry was driven by the need of the Institute for Radiology at Campus Neuromed of the Medical University Linz to collect and analyze the medical outcome data of their patients, who have cerebral aneurysms. The main research subjects of the database are the clinical and morphological follow-ups of patients with cerebral aneurysms, who were treated with an endovascular procedure—either surgically or conservatively.

#### Definition of cerebral aneurysm

A cerebral aneurysm is the dilation, ballooning-out, or bulging of part of the wall of an artery in the brain. Cerebral aneurysms can occur at any age, although they are more common in adults than in children, and are slightly more common in women than in men. The signs and symptoms of an unruptured cerebral aneurysm will partly depend on its size and rate of growth. For example, a small, unchanging aneurysm will generally produce no symptoms, whereas a larger aneurysm that is steadily growing may produce symptoms such as loss of feeling in the face or problems with the eyes. Immediately after an aneurysm ruptures, an individual may experience such symptoms as a sudden and unusually severe headache, nausea, vision impairment, vomiting, and loss of consciousness, leading to a mortality rate of up to 50 % in severe cases. [38]

#### Epidemiological aspects

Intracranial aneurysms occur in the range between 1 and 5 % of the adult population, which accounts for about 12 million patients in the US. Most of these aneurysms (50–80 %) are rather small and do not rupture during a patient’s life time. The estimated incidence rate of subarachnoidal hemorrhage (SAH) from a ruptured intracranial aneurysm is 1 case per 10,000 persons (in the US). SAH is more common in women than in men (2:1), and the peak incidence is found in persons aged 55–60 years. Although the causes of intracranial aneurysms are not known yet, genetic factors may play a role in the development of aneurysms [39, 40].

#### Clinical issues

In the course of this evaluation, we will try to verify the algorithms and their ontology-guided implementation by means of checking them against established medical knowledge and experience from the domain-experts. The medical experts identified a number of features that are known to have an impact on the risk of rupture of a cerebral aneurysm, which are as follows:

- The size of the aneurysm
- The localization of the aneurysm
- The existence of multiple aneurysms
- The age of the patient
- The sex of the patient

We will try to verify this set of influence factors using the proposed ontology-guided meta-classification algorithm and use the ontology-guided dimensionality-reduction algorithm for exploratory data analysis. It is not the intention of this paper to reveal the newly gained medical knowledge, but to verify the gained output of the domain-expert-operated, ontology-guided exploration algorithms against the already known medical evidence.

## Results

The tests were performed by the medical domain-experts on the research data of 1032 cerebral aneurysms belonging to 774 patients. 397 of these aneurysms were ruptured, representing an incidence rate of 38 %.

### Meta-classification

The medical research team tried a number of combinations of potentially influential parameters, and the results were later on discussed. For the first run of the meta-classification algorithm, the following target class was chosen:

- *Aneurysm.Ruptured* This indicates whether the aneurysm is ruptured and can cause a subarachnoid hemorrhage.

As potential input features, the following where selected:

- *Aneurysm.Presentation* Defines how the patient was originally presented at hospital admission. Possible values are Epilepsy, Follow-up, SAH (subarachnoid hemorrhage), and Coincidental.
- *Aneurysm.Location* The anatomic location of the aneurysm with the cerebral vessel structure.
- *Aneurysm.Width* The longest possible diameter throughout the aneurysm volume, which is usually measured via digital substraction angiography (DSA) and 3D volume reconstruction.
- *Patient.Number of Aneurysms* The number of aneurysms the patient has in total.
- *Patient.Age* The age of the patient at the diagnosis date of the aneurysm.

For the first test run, the results are shown in Table 1.

**Table 1:** The area under the ROC curve for the best configuration of each algorithm for the features: Aneurysm.Presentation, Aneurysm.Location, Aneurysm.Width, Patient.Number of Aneurysms, and Patient.Age

NB	RF	MLP	LR	SVM
0.987	0.992	0.988	0.984	0.994

*NB* Naive Bayes,* RF* Random Forest,* MLP*Multi-Layer Perceptron,* LR* Logistic Regression,* SVM* Support Vector Machine

Table 1 shows very promising results at a first glance, but they have to be seen very critically from a medical point of view. The variable *Aneurysm.Presentation* was part of the feature set. This variable contains the value *SAH,* which indicates that the aneurysm is already ruptured. Consequently, when we have an aneurysm which was presented with SAH, then it is absolutely clear that it had already ruptured. Nonetheless, this is a good example that even primitive, linear straight-forward influences can be reliably detected with this method.

In the second mapping, the feature *Aneurysm Presentation* was omitted, in order to investigate how the other parameters influence the risk of rupture. The results are shown in Table 2.

**Table 2:** The areas under the ROC curves for the best configuration of each algorithm for the features: Aneurysm.Location, Aneurysm.Width, Patient.Number of Aneurysms, and Patient.Age

NB	RF	MLP	LR	SVM
0.779	0.814	0.776	0.793	0.809

*NB* Naive Bayes,* RF* Random Forest,* MLP * Multi-Layer Perceptron,* LR* Logistic Regression, SVM - Support Vector Machine

The areas under the ROC curve for all algorithms are significantly lower than for the first feature set. However, an obvious distance from the 0.5 area remains, indicating the influences of these features on the risk of rupture, the same as those that the medical experience confirms. In a further step, the set of features was extended by the sex of the patient. The results are shown in Table 3.

**Table 3:** The areas under the ROC curves for the best configuration of each algorithm: Aneurysm.Location, Aneurysm.Width, Patient.Number of Aneurysms, Patient.Age, and Patient.Sex

NB	RF	MLP	LR	SVM
0.789	0.820	0.773	0.800	0.812

*NB* Naive Bayes,* RF* Random Forest,* MLP* Multi-Layer Perceptron,* LR* Logistic Regression,* SVM* Support Vector Machine

Here, only a minimal improvement compared to Table 2 could be observed.

### Dimensionality reduction

The visual clustering algorithms were applied to the same datasets using the same feature set as described in Sect. 5.1. Furthermore, the visualizations aim to show the related correlations and patterns that were already identified by the ontology-guided meta-classification.

**Fig. 2:**
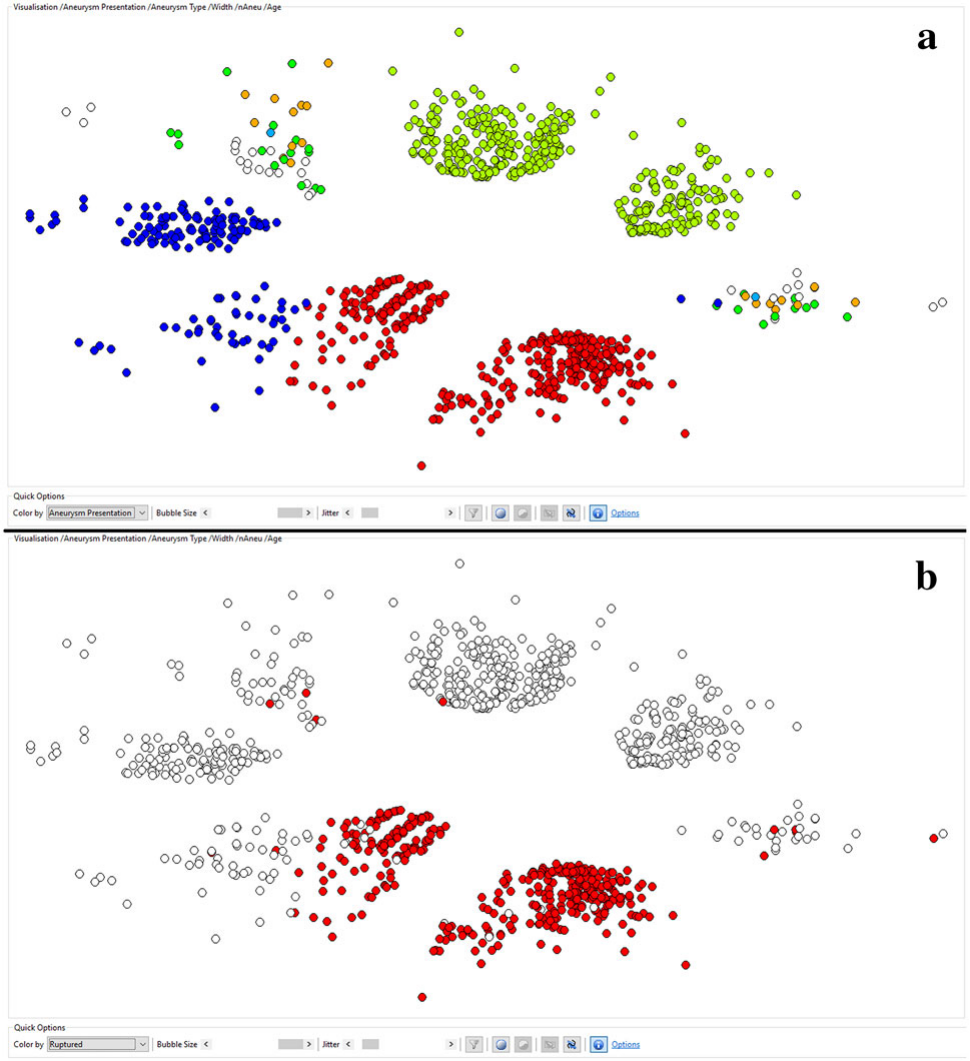
An ontology-guided nonlinear mapping of 1032 cerebral aneurysms with a distance calculation based on the following features: Aneurysm.Presentation, Aneurysm.Width, Aneurysm.Location, Patient.Number of Aneurysms, and Patient.Age. **a** The aneurysms are colored according to their presentation: * green* is incidental, * blue* is coincidental, and red is after a subarachnoid bleeding. **b** The aneurysms are colored according to their rupture state * red* are ruptured, * white* are non-ruptured. (Color figure online)

Figure 2 shows the results for the first mapping. The distance of the aneurysms in the high-dimensional source space was calculated based on the following features: *Aneurysm.Presentation*, *Aneurysm*. *Location*, *Aneurysm*. *Width*, *Patient.Number of Aneurysms*, and *Patient.Age*. Figure 2 contains two screenshots directly taken from the ontology-centered research infrastructure. The first one (*a*) is colored according to the presentation type of the aneurysm, and the latter one (*b*) is colored by the rupture state. The visualization echoes the impression that we gained from the tests with the meta-classifier. Once the aneurysm presentation type is known, the prediction of the rupture state of the aneurysm is possible with a very high reliability.

**Fig. 3:**
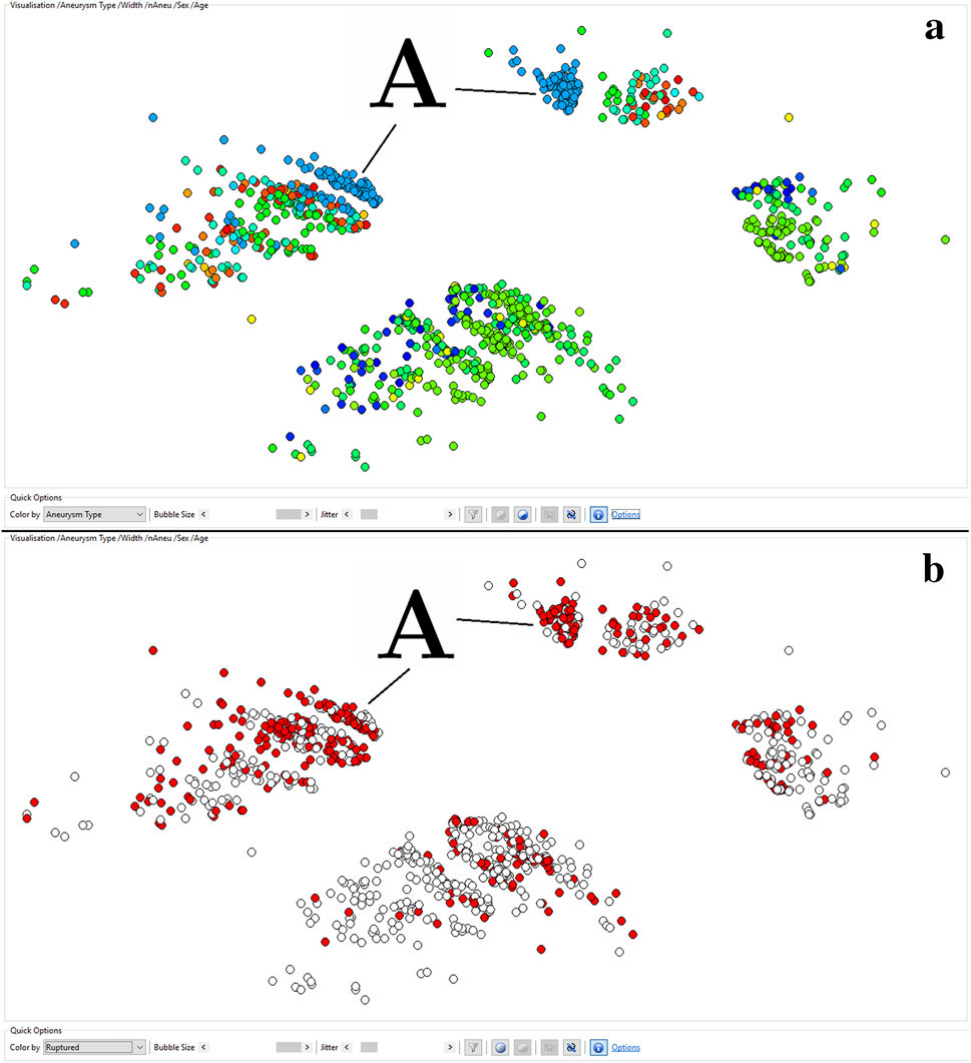
An ontology-guided nonlinear mapping of 1032 cerebral aneurysms with a distance calculation based on the following features: Aneurysm.Width, Aneurysm.Location, Patient.Number of Aneurysms, and Patient.Age. **a** The aneurysms that are colored according to their location. **b** The aneurysms that are colored according to their rupture state * red* are ruptured, * white* are non-ruptured. (Color figure online)

In accordance with the tests in Sect. 5.1, a second mapping was created by omitting the variable *Aneurysm*. *Presentation* but taking into account the sex of the patient. The results of this visual clustering are shown in Fig. 3.

The distance of the aneurysms in the high-dimensional source space was calculated based on the following features: *Aneurysm.Width*, *Aneurysm. Location*, *Patient.Number of Aneurysms*, *Patient.Age*, and *Patient.Sex*. The general structure of the shows four clearly separable clusters, while the two clusters on the left-hand side show female patients and the clusters on the right-hand side show male patients. The size difference reflects the fact that about two-thirds of aneurysm patients are female. The horizontal separation is caused by the location of the aneurysm. The northern clusters contain aneurysms, located in the anterior circulation of the brain. The southern area contains aneurysms of the posterior circulation. The dots in the upper plot (*a*) are colored according to the exact location of the aneurysm. The dots in the lower plot (*b*) are again colored by the rupture state of the aneurysms. In comparison with the section (*b*) of Fig. 2, the separation between the ruptured and the non-ruptured aneurysms is not that clear anymore. However, there are still areas with higher densities of red dots and areas where this density is remarkably lower, which is in accordance with the results that were observed in Sect. 5.1, when the Aneurysm.Presentation was removed as a feature. Remarkable in section (*b*) of Fig. 3 are the two very dense red (ruptured) areas which are marked with a capital *A*. The same areas are marked in section (*a*) of the same figure. It immediately strikes that both areas are marked with the same color, meaning they share the same location. The separation between these two clusters is caused by the split by Patient.Sex, which was also a feature in this case. The right-hand side aneurysms are from male patients and the left ones from females. The said location is the Anterior Communicating area, which is known to have a higher risk for aneurysm rupture [41].

## Discussion

All the known and relevant process models for knowledge discovery find the (medical) domain-expert in a customer-like, supervising role [3, 42]. While the scientific community is slowly realizing what benefits can be gained when the domain-expert is deeply integrated into the data-mining and machine-learning loop, no relevant research on the knowledge-discovery process could be found.

We proposed a new process model for expert-driven knowledge discovery in medical research. It eliminates the frequent tasks, *Understanding the Domain* and *Understanding the Data*, from the known models and replaces these tasks by the following tasks: *Data Modeling*, *Data Acquisition*, and *Data Validation*. For the software support of this new process model, an ontology-centered approach was chosen. In the first step of the new process (Data Modeling), the domain-experts define what data (structures) are necessary for the current research questions to be answered. This definition is stored in the form of a domain ontology, which is subsequently used to actively support the user in all the tasks of the process.

In this paper, we demonstrated how the extensive use of ontology-originated, structural meta-information can help the medical domain-expert to familiarize himself with the application of advanced machine-learning and visualization algorithms—algorithms that are usually the preserved domain for the IT and machine-learning experts. By automatizing the data pre-processing and algorithm parametrization to a very high degree, it is possible even for a non-IT user to apply these algorithms and find answers to their research questions. The visual-analysis algorithms were able to detect a correlation between the risk of rupture and the location of the aneurysm, which was already medically evident.
